# Supplementary material for: Application of Near-Infrared Spectroscopy to Investigate Some Endogenic Properties of Pleurotus ostreatus Cultivars
Source: Sensors (Basel). 2020 Nov 19;20(22):6632. doi: 10.3390/s20226632 (PMC7699429; doi:10.3390/s20226632)
Supplement: Supplementary file 1 [file sensors-20-06632-s001.pdf]

Supplementary Materials

Table S1. Total free amino acid (fAA) concentration of cultivars.

**Cultivars**	**Total fAA g/100g**	**Cultivars**	**Total fAA g/100g**
Po_1	10.1 ± 0.4	Po_8	8.2 ± 0.1
Po_2	13.6 ± 0.4	Po_9	8.9 ± 0.3
Po_3	12.1 ± 0.2	Po_10	7.0 ± 0.2
Po_4	10.0 ± 0.3	Po_11	9.9 ± 0.2
Po_5	8.9 ± 0.2	Po_12	10.3 ± 0.2
Po_6	10.6 ± 0.2	Po_13	9.0 ± 0.3
Po_7	6.5 ± 0.3	Po_14	7.8 ± 0.2

Table S2. Free amino acid concentration (mg/100g).

Cultivars	Po_1	Po_2	Po_3	Po_4	Po_5	Po_6	Po_7	Po_8	Po_9	Po_10	Po_11	Po_12	Po_13	Po_14
Asp	504±41.3	513±22.6	359±28.3	410±29.2	380±44.6	383±20.0	339±28.2	168±5.3	405±8.0	228±23.7	334±24.0	370±18.0	372±13.5	211±17.5
Thr	446±22.7	652±59.3	420±3.7	383±21.9	388±23.5	461±41.0	318±19.3	200±11.3	357±14.0	132±7.5	353±1.7	377±19.6	363±25.4	271±6.6
Ser	431±1.0	576±16.7	420±19.8	356±1.2	365±16.8	382±12.2	307±12.7	234±17.2	333±13.4	130±15.3	333±36.	371±17.8	336±18.5	223±25.0
Asn	974±60	1733±214	2425±354	1836±101	1769±86	1301±305	780±39	2574±173	2345±403	2781±393	3101±165	2532±274	1627±2	3427±119
Glu	1388±123	1810±183	2076±162	1333±103	1093±20	1462±40	1069±105	1600±70	964±114	1416±270	821±61	1212±144	1150±94	491±10
Gln	170.2±39.9	112.2±19.9	210.4±73.2	117.6±63.3	68.0±6.7	266.1±13.1	78.3±6.7	161.9±13.7	62.8±12.0	153.2±19.1	38.9±5.3	90.8±2.5	65.3±6.2	30.5±6.5
Pro	426.9±45.2	750.7±126	336.4±21.1	449.4±30.0	456.0±39.0	423.0±40.0	316.9±19.2	159.5±11.0	385.9±47.4	68.1±2.8	395.9±60.9	450.1±23.6	319.3±7.0	247.6±33.5
Gly	256.2±33.9	342.6±39.2	223.1±19.3	235.7±23.2	187.9±11.2	214.6±12.5	156.3±10.4	104.0±4.8	211.2±6.	55.6±3.9	196.1±4.0	231.9±5.1	188.9±7.1	124.4±6.6
Ala	777±14.6	1173±59.2	855±52.2	868±87.7	740±59.7	777±28.6	546.3±34.1	358.4±16.6	680±52.7	231.1±5.7	710±4.4	968±71.1	773±47.2	478.1±14.4
Val	602±6.2	739±25.2	437.3±52.5	423.8±51.1	441.6±29.0	472.7±20.0	300.7±11.4	153.5±13.9	399.5±36.8	69.7±11.8	461.0±26.0	462.2±61.6	447.8±71.5	296.4±41.1
Cys	< DL	33.5±6.1	< DL	51.4±7.3	59.8±2.9	49.3±6.3	< DL	8.0±0.8	< DL	< DL	22.3±4.0	< DL	15.2±2.5	< DL
Met	139.8±6.0	216.3±7.0	121.4±20.9	121.3±8.3	113.6±3.8	141.1±13.3	105.0 ±6.3	33.9±9.4	59.7±11.5	27.3±3.3	96.7±13.5	82.3±16.0	117.5±8.	51.8±2.7
Ile	385.1±17.3	485.3±49.2	267.1±39.9	357.0±43.5	267.1±9.6	334.6±8.6	201.9±36.3	118.9±14.4	279.7±10.8	84.4±10.1	306.0±12.5	312.5±11.7	318.1±13.5	202.9±9.6
Leu	846±.46.3	1066±59.8	633±32.8	716±9.5	574±18.3	820±37.0	479.8±16.6	205.2±9.8	395.5±5.6	82.9±2.2	634±41.5	609±35.5	641±31.3	406.4±38.4
Tyr	410.8±19.5	462.0±16.8	324.4±34.7	321.4±46.4	257.3±25.6	399.9±30.7	194.8±13.7	122.7±9.9	256.8±10.7	95.1±9.8	285.0±29.2	329.5±28.3	301.6±31.3	188.5±18.2
Phe	387.1±48.7	569.3±30.4	292.3±10.5	273.2±33.9	240.6±29.5	444.2±32.9	239.7±22.2	108.6±11.7	243.9±25.6	73.3±8.9	284.6±39.5	277.2±19.6	323.0±39.0	190.6±15.2
Ornitin	313.5±40.1	437.9±90.3	1022±106	532.2±62.6	313.4±70.7	280.6±89.5	256.8±66.1	1128±204	445.3±30.9	762±27.0	343.4±7.9	530.1±21.6	358.2±13.7	206.2±49.3
Lys	123.7±8.3	94.2±14.3	110.8±3.8	40.4±4.6	45.5±8.6	144.5±18.7	34.5±10.1	41.3±8.6	66.1±6.4	42.3±3.8	65.2±9.3	80.9±8.1	83.3±6.5	40.7±5.4
His	552.0±28.4	694.6±6.3	369.9±8.7	347.4±38.1	339.7±19.9	528.9±19.8	253.9±11.2	145.3±6.5	300.9±12.4	80.2±3.5	354.5±43.2	316.4±22.7	331.5±27.6	227.8±19.8

Arg	590.7±58.0	719±59.6	671±69.4	445.5±32.4	462.6±31.1	893±34.4	249.8±16.3	357.5±51.6	373.2±14.5	313.2±1.3	456.6±45.7	366.9±10.4	540.0±48.7	325.0±28.6
GABA	97.7±13.2	104.2±16.0	85.6±5.5	115.6±14.2	105.2±2.5	99.5±14.8	83.5±10.2	67.9±6.4	105.4±11.3	44.6±2.7	88.9±27.5	70.0±3.6	83.3±4.2	37.8±2.5
Cisztationin	49.0±3.4	43.9±3.6	185.8±2.6	92.2±2.1	120.9±4.1	87.8±5.9	71.9±15.0	26.4±3.6	59.1±3.6	28.7±9.3	80.6±10.3	87.2±10.6	64.0±15.7	32.2±6.7
HME	228.1±19.5	301.3±23.5	265.4±34.7	203.9±11.7	151.8±16.4	221.5±25.9	121.9±12.2	152.4±17.3	142.0±23.1	121.4±1.9	158.0±14.9	182.2±16.3	176.9±16.0	113.7±6.1

DL detection limit (DL_Cys = 1μg/100g)

Table S3. Results of Kruskal-Wallis test. Multiple pairwise comparisons for free total amino acid.

Cultivar	Sum of ranks	Mean of ranks	Groups							
Po_7	6.000	2.000	A							
Po_10	15.000	5.000	A	B						
Po_14	24.000	8.000	A	B	C					
Po_8	33.000	11.000		B	C	D				
Po_9	49.000	16.333			C	D				
Po_5	51.000	17.000				D	E			
Po_13	53.000	17.667				D	E			
Po_11	76.000	25.333					E	F		
Po_1	84.000	28.000						F		
Po_4	81.000	27.000						F		
Po_12	93.000	31.000						F	G	
Po_6	101.000	33.667						F	G	H
Po_3	114.000	38.000							G	H
Po_2	123.000	41.000								H

Table S4. Results of Kruskal-Wallis test. Multiple pairwise comparisons for cysteine concentration.

Sample	Sum of ranks	Mean of ranks	Groups					
Po_1	33.000	11.000	A					
Po_3	33.000	11.000	A					
Po_7	33.000	11.000	A					
Po_9	33.000	11.000	A					
Po_10	33.000	11.000	A					
Po_12	33.000	11.000	A					
Po_14	33.000	11.000	A					
Po_8	69.000	23.000		B				
Po_13	78.000	26.000		B	C			
Po_11	87.000	29.000			C	D		
Po_2	96.000	32.000				D		
Po_6	108.000	36.000					E	
Po_4	112.000	37.333					E	F
Po_5	122.000	40.667						F

Table S5. Results of Kruskal-Wallis test. Multiple pairwise comparisons for methionine concentration.

Sample	Sum of ranks	Mean of ranks	Groups					
Po_10	10.000	3.333	A					
Po_8	11.000	3.667	A					
Po_14	27.000	9.000	A	B				
Po_9	31.000	10.333	A	B				
Po_12	44.000	14.667	A	B	C			
Po_11	53.000	17.667		B	C	D		
Po_7	63.000	21.000		B	C	D		
Po_5	73.000	24.333			C	D	E	
Po_13	82.000	27.333				D	E	
Po_3	85.000	28.333				D	E	
Po_4	86.000	28.667				D	E	
Po_6	107.000	35.667					E	F
Po_1	108.000	36.000					E	F
Po_2	123.000	41.000						F

Table S6. Total polyphenol content (µmol GAE/g).

Cultivars	TPC µmol GAE/g	Cultivars	TPC µmol GAE/g
Po_1	66.31 ± 1.70	Po_8	67.96 ± 0.66
Po_2	114.43 ± 2.20	Po_9	87.55 ± 0.83
Po_3	85.06 ± 1.19	Po_10	65.64 ± 0.91
Po_4	82.60 ± 1.72	Po_11	89.15 ± 1.43
Po_5	175.58 ± 0.87	Po_12	90.54 ± 2.08
Po_6	147.05 ± 1.98	Po_13	108.15 ± 7.83
Po_7	120.79 ± 2.16	Po_14	108.15 ± 1.68

Table S7. Results of Kruskal-Wallis test. Multiple pairwise comparisons for total polyphenol content.

Cultivar	Sum of ranks	Mean of ranks	Groups								
Po_10	36.000	6.000	A								
Po_1	49.000	8.167	A	B							
Po_8	86.000	14.333		B							
Po_4	132.000	22.000			C						
Po_3	163.000	27.167			C						
Po_9	208.000	34.667				D					
Po_11	240.000	40.000				D	E				
Po_12	262.000	43.667					E				
Po_13	491.000	54.556						F			
Po_14	349.000	58.167						F			
Po_2	399.000	66.500							G		
Po_7	435.000	72.500							G	H	
Po_6	471.000	78.500								H	I
Po_5	507.000	84.500									I

Table S8. FRAP values (µmol AAE/g) of cultivars.

Cultivars	FRAP. µmol AAE/g	Cultivars	FRAP. µmol AAE/g
Po_1	8.88 ± 0.12	Po_8	11.00 ± 0.65
Po_2	20.27 ± 0.16	Po_9	14.40 ± 0.83
Po_3	9.20 ± 0.35	Po_10	5.74 ± 0.28
Po_4	13.89 ± 0.31	Po_11	15.81 ± 0.71
Po_5	20.68 ± 0.85	Po_12	16.31 ± 0.57
Po_6	11.68 ± 0.30	Po_13	18.38 ± 0.50
Po_7	12.93 ± 0.48	Po_14	7.83 ± 0.76

Table S9. Multiple pairwise comparisons for FRAP values.

Cultivar	Sum of ranks	Mean of ranks	Groups							
Po_10	21.000	3.500	A							
Po_14	61.000	10.167	A							
Po_1	117.000	19.500	A	B						
Po_3	136.000	22.667	A	B	C					
Po_8	190.000	31.667		B	C	D				
Po_6	212.000	35.333		B	C	D	E			
Po_7	255.000	42.500			C	D	E	F		
Po_4	302.000	50.333				D	E	F	G	
Po_13	478.000	53.111					E	F	G	
Po_9	319.000	53.167					E	F	G	
Po_11	369.000	61.500						F	G	H
Po_12	390.000	65.000							G	H
Po_2	487.000	81.167								H
Po_5	491.000	81.833								H
